# Short Term Survival after Admission for Heart Failure in Sweden: Applying Multilevel Analyses of Discriminatory Accuracy to Evaluate Institutional Performance

**DOI:** 10.1371/journal.pone.0148187

**Published:** 2016-02-03

**Authors:** Nermin Ghith, Philippe Wagner, Anne Frølich, Juan Merlo

**Affiliations:** 1 Unit for Social Epidemiology, Faculty of Medicine, Lund University, Malmö, Sweden; 2 Research Unit of Chronic Conditions, Bispebjerg University Hospital, Copenhagen, Denmark; 3 Centre for Clinical Research, Västmanland, Uppsala University, Västerås, Sweden; IIBB-CSIC-IDIBAPS, SPAIN

## Abstract

**Background:**

Hospital performance is frequently evaluated by analyzing differences between hospital averages in some quality indicators. The results are often expressed as quality charts of hospital variance (e.g., league tables, funnel plots). However, those analyses seldom consider patients heterogeneity around averages, which is of fundamental relevance for a correct evaluation. Therefore, we apply an innovative methodology based on measures of components of variance and discriminatory accuracy to analyze 30-day mortality after hospital discharge with a diagnosis of Heart Failure (HF) in Sweden.

**Methods:**

We analyzed 36,943 patients aged 45–80 treated in 565 wards at 71 hospitals during 2007–2009. We applied single and multilevel logistic regression analyses to calculate the odds ratios and the area under the receiver-operating characteristic (AUC). We evaluated general hospital and ward effects by quantifying the intra-class correlation coefficient (ICC) and the increment in the AUC obtained by adding random effects in a multilevel regression analysis (MLRA). Finally, the Odds Ratios (ORs) for specific ward and hospital characteristics were interpreted jointly with the proportional change in variance (PCV) and the proportion of ORs in the opposite direction (POOR).

**Findings:**

Overall, the average 30-day mortality was 9%. Using only patient information on age and previous hospitalizations for different diseases we obtained an AUC = 0.727. This value was almost unchanged when adding sex, country of birth as well as hospitals and wards levels. Average mortality was higher in small wards and municipal hospitals but the POOR values were 15% and 16% respectively.

**Conclusions:**

Swedish wards and hospitals in general performed homogeneously well, resulting in a low 30-day mortality rate after HF. In our study, knowledge on a patient’s previous hospitalizations was the best predictor of 30-day mortality, and this information did not improve by knowing the sex and country of birth of the patient or where the patient was treated.

## Introduction

Heart Failure (HF) is a serious, life-threatening condition that causes considerable disability. However, early diagnosis and adequate treatment for heart failure can improve life quality and prolong survival.[1]

Short term mortality within 30 days after a discharge diagnosis of heart failure is a commonly used quality indicator for evaluating hospital performance [2–5] which, as any other quality indicator, requires regular assessment.

Nowadays, multilevel regression analysis (MLRA) is becoming established as a suitable methodology for the evaluation of institutional performance [6, 7]. MLRA takes into account the multilevel structure of the data (e.g., patients nested within hospitals). This technique allows a less biased estimation of uncertainty providing a better ranking of the health care units under investigation. In addition, MLRA allows a flexible analysis of components of variance and permits estimating measures of association at different levels of analysis [8–11]. Nevertheless, many studies performed today are still using single level designs analyzing, for instance, individual patient data with dummy variables for the hospitals [8], or information aggregated at the hospital level [12–15]. Even when high quality data is available at the patient, institutional and geographical levels, assessments are normally performed at one level by using, for instance, funnel plots [16], health league tables [17], or similar [18] [19] to compare hospitals or small geographical area averages [20, 21]. This single level approach is extensively used, but may provide misleading information for decision makers [22].

Conceptually, most studies evaluating institutional performance in health care (e.g., hospitals) make two implicit assumptions. First, it is assumed that over and above patient characteristics, the hospital context exerts a general, shared effect on all patients at the hospital. The existence of general, unspecified institutional effects originates differences between institutions that condition individual prognosis over and above individual characteristics. However, while the hospital level itself may play a direct role, most of this type of influence could result at the ward level within the hospitals where the patients are actually treated.

Second, it is often assumed that this general institutional effect can be measured by quantifying differences between averages in certain quality indicators. Here, researchers may perform funnel plots, control charts or ‘league tables’ where, for instance, hospitals are ranked according to their average 30-day mortality after admission for heart failure. However, for evaluating general institutional effect, what matters mostly is not the existence of differences between hospital averages, but rather the share of the patient differences in the outcome at the hospital level. This concept corresponds nicely with the idea of intra-class correlation (ICC) used to quantify general contextual effects in social epidemiology [23, 24], and it has been previously applied for assessing institutional performance [10, 25, 26]. This idea is even closely related to the notion of discriminatory accuracy (DA) developed for the evaluation of the performance of prognostic and screening markers in medicine [27, 28]. It is therefore possible to apply MLRA and use measures of DA like the area under the Receiving Operator Characteristics curve (AUC) to quantify general hospital effects [29–31]. In contrast with the ICC for binary outcomes [32], measures of DA like the AUC are well established in clinical and health care epidemiology and the information they give is relatively easy to interpret and communicate.

The application of AUC measures is also convenient as the evaluation of hospital performance (e.g., league tables) is often used as a basis for informed decisions. In this aspect, the evaluation of hospital performance resembles a screening test so we should know the discriminatory accuracy of, for instance, a funnel plot or a hospital league table before we use it to make decisions.

With the above background, our study quantifies to which extent hospital and ward differences in averages are relevant for understanding patient disparities in short term mortality after heart failure in Sweden. For this purpose, we perform a multilevel analysis that distinguishes between hospital, ward and patient components of variance and informs on both general and specific contextual effects on patient survival. We also apply a novel methodological approach based on measures of discriminatory accuracy [29–31]. In this approach, we considered information on hospitals and wards (i.e., league tables) as a tool for classifying patients according to their 30-day survival and assess the AUC of this instrument over and above patients’ characteristics.

## Population and Methods

### Study population

The National Board of Health and Welfare linked information from the Swedish Patient Register and the Cause of Death Register using a unique personal identification number. This data was then linked to the Longitudinal Integrated Database for Health Insurance and Labour Studies (LISA) that is maintained by Statistics Sweden and contains demographic and socioeconomic information. Finally, the Swedish authorities delivered the research database to us without the personal identification numbers to ensure the anonymity of the subjects.

From the research database we identified all 60,130 patients with a discharge diagnosis of heart failure (International Classification of Diseases code I50) admitted to Swedish hospitals between 2007–2009 with an age between 45 to 80 years. A major problem when comparing differences in outcome indicators like mortality is the threat of confounding by patient-mix. That is, some hospitals may provide specialized care to patients with a higher degree of co-morbidity than others and, in turn, co-morbidity is associated to mortality. Since previous heart failure is a strong determinant of mortality after a new hospitalization for heart failure, the most appropriate way of evaluating hospital quality in relation to survival after heart failure is to observe survival in patients with a first diagnosis of heart failure. In this way, we increase the homogeneity of this patient group, which gives a better basis for less confounded analyses. Therefore, we excluded 22,587 patients with a previous diagnosis of heart failure in the past five years.

**Fig 1:**
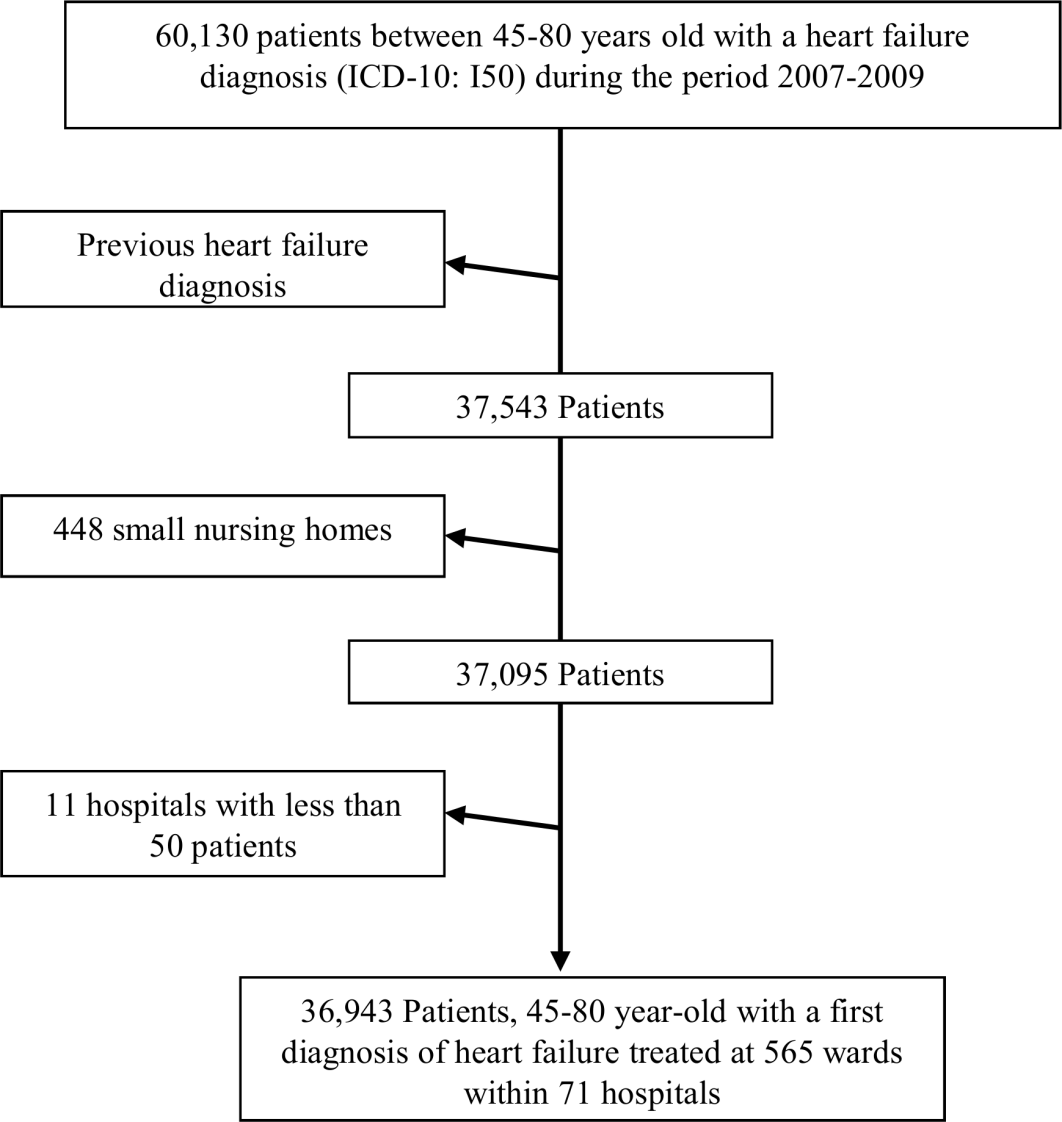
Study Population. Flow diagram showing the selection of patients with first diagnosis of heart failure between 2007 and2009 who were included in the study population.

Finally, as our focus was to evaluate hospitals, we excluded 448 patients treated at small nursing homes. In addition, to ensure the stability of the estimations, we arbitrarily excluded hospitals with less than 50 patients that led to a further exclusion of 152 patients. The final data set included 36, 943 patients within 565 wards from 71 hospitals (Fig 1).

### Assessment of Variables

#### Patient outcome

The study outcome is all-cause mortality within 30-days after discharge from the hospital (coded yes vs. no).

#### Patient characteristics

Since survival after heart failure may be conditioned by the gender and ethnic origin of the patient, we created a combined variable with four categories: Swedish females, Swedish males, non-Swedish females, and non-Swedish males and used the Swedish females as the reference group in subsequent comparisons.

An intrinsic problem when analyzing outcome indicators like mortality is the peril of selection bias so patients with a worse prognosis are channeled to certain hospitals. Therefore, hospital differences may be confounded by this selection of patients. Therefore, besides excluding patients with a previous diagnosis of HC, to reduce this compositional confounding (i.e., case-mix), we computed a risk score (RS) for 30-days mortality using previous discharge diagnosis (see methods section). We categorized the RS into 10 groups by deciles, using the first decile group as the reference in the comparisons.

#### Ward and hospitals characteristics

Results from previous studies [2, 3, 33, 34] suggest that, on average, the prognosis of patients with heart failure improves when they are treated at institutions with higher volumes of patients. This situation could be explained by the fact that the medical staff could have more expertise. It may also originate in a selective referral of patients to hospitals with good reputation which causes higher volumes of patients [35]. This selective referral might in turn channel patients with more severe conditions and act as a confounder that impairs average mortality. The exclusive use of administrative data for volume–outcomes research may, therefore, generate spurious findings. [36] Additionally, when studying volume of patients as a contextual variable rather than the hospital as a whole, the relevant level of analysis should be the ward where the patients are actually treated.

Aware of those difficulties, we computed a variable informing the number of patients with heart failure admitted to each hospital ward. We then categorized this variable into three groups by tertiles, using the first tertile group as the reference in the comparisons.

We also categorized the hospitals according to the traditional Swedish classification as (i) regional, (ii) provincial and (iii) municipal since the type of hospital could have an impact on mortality over and above ward and patient characteristics.

### Statistical Methods

#### Risk score (RS) for mortality

Using a single level stepwise backward logistic regression, we modeled 30-days mortality as a function of age and previous diseases (ICD-10 codes) and obtained the predicted probability (i.e., individual risk score) of 30-days mortality. Table 1 shows the out- and inpatients diagnoses considered in an initial stepwise logistic regression to develop the RS equation. The purpose for developing the RS was not to create an equation for future prediction of 30-days mortality for patient with heart failure [37] as those currently available for patient-mix adjustment (see, for instance, the Charlson Risk Score [38], The Elixhauser Score [39], and the CMS Hierarchical Condition Category "CMS-HCC" risk adjuster [40]). Rather, our aim was to perform a parsimonious analysis where the RS summarizes a large number of variables into a single construct. Otherwise, the adjusted models using the RS gave the same results as a model including the disease variables separately used for the computation of the RS.

**Table 1:** Characteristics of patients (N = 36,943) with first ever diagnosis of heart failure who were admitted to Swedish hospitals during 2007–2009 (Values are percentages if not otherwise indicated).

	Hospital classification		
	Municipal	Provincial	Regional
30-day Mortality	8.2	9.4	9.1
Patients with heart failure	29.0	42.5	28.5
Number of hospitals	41	21	9
Number of patients with heart failure at the hospital *Median (min-max)*	536 (188–2069)	1258 (602–2841)	2414(1371–4186)
Number of wards	163	198	204
Number of patients with heart failure at the ward. *Median (min-max)*	237 (1–901)	445 (1–968)	313 (1–895)
Age mean (SD)	71 (8)	71 (8)	70 (8)
Gender by Ethnic origin			
- Swedish males	52.1	51.9	47.4
- Swedish females	34.9	33.9	32.8
- Non Swedish males	6.9	8.0	11.5
- Non Swedish females	6.1	6.2	8.3
Risk Score*, mean	0.089	0.089	0.091
Risk Score, decile groups			
- 1^st^	9.3	9.6	10.3
- 2^nd^	10.1	9.9	9.4
- 3^rd^	12.0	12.1	11.3
- 4^th^	9.7	9.1	8.8
- 5^th^	10.4	10.3	10.4
- 6^th^	9.9	9.1	9.2
- 7^th^	10.5	10.3	10.7
- 8^th^	9.2	9.6	9.5
- 9^th^	9.3	10.2	9.7
- 10^th^	9.6	9.7	10.7
Volume of patients with heart failure at wards (Tertiles)			
- First tertile	43.8	21.6	41.4
- Second tertile	47.7	24.3	34.7
- Third tertile	8.4	54.1	23.9

*Risk Score obtained from a logistic regression including age, previous diagnoses (ICD-10) of diseases of the cerebral arteries (I6), arrhythmia (I48-I49), hypertension (I10-I13&I15), ischemic coronary artery disease (I20-I25), varicose (I83), peripheral vascular disease (I74&I80), acute myocardial infarction (I21), other types of heart disease (I3-I5), respiratory diseases (J0-J9), digestive diseases (K0-K9), diabetes (E10-E14), infectious diseases (A0-A9), cancer (C1-D4), lung cancer (C34), chronic diseases of the lower respiratory tract (J4), immunity disorder (D50-D89), mental diseases (F0-F9), and injury (S00-T14).

#### Regression models analyses

We applied an original, stepwise-multilevel, logistic regression analysis of discriminatory accuracy recently developed by this team [31]. We used 36,943 patients nested within 565 wards that were, in turn, nested within 71 hospitals. We developed four consecutive logistic regression analyses to model mortality.

For every model equation, we obtained the predicted logit and computed *the area under the receiver operator characteristics (AUC) curve or C statistic*. The AUC measures the ability of the model to correctly classify individuals with or without the outcome (e.g., mortality within 30 days). The AUC assumes a value between 1 and 0.5 where 1 is perfect discrimination and 0.5 would indicate that the model is as informative as flipping a coin (i.e., the covariates have no predictive power) [28].

In the first model (*model 1*), we wanted to evaluate the influence of patients’ characteristics alone on short term mortality. We fitted a single level logistic regression model including only the risk score for mortality (RS). We obtained the AUC as a measure of the capacity of the individual level information (i.e. RS) to classify with accuracy the patients who survive from those who die.

In the second model (*model 2*), we added the combined explanatory variable of gender by migration/ethnic status. We aimed to know the value added of this information to classify with accuracy the patients who survive from those who die over and above the RS for mortality. For this purpose, we calculated the AUC and the difference between the AUC value of model 2 and 1.

In the next model (*model 3*), we expanded model 2 by including two random intercepts one for the wards inside hospitals and another for the hospitals level in a three level multilevel regression model. We calculated the intra-class correlation coefficient for the hospital (ICC_h)_ and for the ward level (ICC_w_) from this model.

The ICC measures the observational general hospital/ward effect. Expressed as a percentage, the value of the ICC goes from 0% to 100%. If the hospitals/wards were not relevant for understanding patient short term mortality differences in Sweden, the ICC_h_ would be close to 0%. That is, the hospitals would be similar to random samples taken from the whole patient population.

As complementary information, we also computed *the median odds ratio (MOR)* [32, 41, 42].

In the absence of variation, the MOR is equal to 1. The higher the MOR, the more relevant the hospital/ward is for understanding the individual outcome.

In the calculation of the AUC for the MLRA models, the prediction equation includes the random effects (i.e., higher level residuals) as has been discussed elsewhere [29–31]. By computing the difference between the AUC of the models 3 and 2, we obtained information on the added value of knowing the hospital and the ward where the patient was treated in order to classify with accuracy the patients who survive from those who die, over and above patient information. This difference also assesses the relevance of the hospitals and the wards for patients’ short term mortality, which provides complementary information to that obtained by the ICC [29, 30]. We also calculated two alternative AUCs in model 3. One was based on an equation that excluded the hospital residuals and another that excluded the wards residuals. In this way, we could quantify the contribution of having only information from one of those two levels.

Furthermore, to illustrate the differences between wards and hospitals averages in short term mortality, we created league tables by ranking hospitals and wards according to their average 30-day mortality rate using the values of the shrunken residuals and their 95% confidence intervals. The value added of using the league tables alone for auditing outcome quality is quantified by the ICC in model 3 and also by the difference between AUCs of model 3 and 2.

In the next model (*model 4)*, we added the ward and the hospital level specific variables indicating the volume of patients with heart failure (in tertile groups) as well as the hospital classification.

Our aim was to understand the mechanism underlying eventual ward and hospital general effects. For this purpose, besides measuring specific contextual effects (i.e., odds ratios), we also calculated the proportional change in variance (PCV) as the percentage of the wards and hospitals variance in model 3 that was explained by the specific wards and hospitals variables in model 4.

$$PCV=\frac{{(\sigma }_{u\{h,w\}}^{2}\;previous\;model\;3-\sigma_{u\{h,w\}}^{2}\;last\;model\;4)}{\sigma_{u\{h,w\}}^{2}\;previous\;model3}*100$$

We calculated AUC for model 4 as well as the difference between the AUC value of model 4 and model 2. We observed that model 4 cannot increase the AUC value obtained in previous model 3. As the inclusion of specific contextual variables as fixed effects will eventually explain some of the wards and hospitals intercept variance (i.e., decrease the shrunken residuals), they simultaneously improve the prediction equation by adding two regression-coefficients for the variables ward volume of patients and hospital classification. That is, the increase in the AUC in model 3 compared to model 2 represents the ceiling of the hospital’s general effect. Assuming an appropriate patient mix adjustment, it represents both measurable and immeasurable institutional factors that condition the prognosis of the HF patients.

We used the Proportion of Opposed Odds Ratios (POOR) for the contextual variable on hospital type. The values of the POOR extend between 0% and 50%. A POOR of 0% means all ORs have the same sign. A POOR of 50% would mean that half of the ORs are of the opposite sign and so the association is very heterogeneous. We calculated the POOR for the hospital and the ward level. See S1 Appendix for specific information.

For the estimation of the models we initially used the Restricted Iterative Generalized Least Squares (RIGLS) method to obtain values for the final Markov Chain Monte Carlo (MCMC) estimation method [43]. We used the Bayesian Deviance Information Criterion (BDIC) as a measure of the goodness of fit of the models [44]. The BDIC considers both the model deviance and its complexity. Models with smaller BDIC should be preferred to models with larger BDIC.

We estimated the variance as the median and 95% credible intervals of the posterior distribution obtained by the Markov Chain Monte Carlo (MCMC) method [43] and included additional technical details on the all the models and measures in S1 Appendix.

We performed the analyses using IBM SPSS Statistics for Windows, Version 21 (IBM Corp., Armonk, NY, USA), STATA, StataCorp. 2013. Stata Statistical Software: Release 13. College Station, TX: StataCorp LP and MLwiN version 2.22, The Centre for Multilevel Modeling, University of Bristol.

### Ethics statement

The Regional Ethics Review Board in southern Sweden (# 2012/637) as well as the data safety committees from the National Board of Health and Welfare and from Statistics Sweden approved the construction of the database.

## Results

### Characteristics of the population

Table 1 shows that most of the patients with heart failure were treated at the 21 provincial hospitals. However, the median number of patients was highest at the nine regional hospitals and lowest at the 41 municipal hospitals. Additionally, wards at the provincial hospitals had the highest volume of patients with heart failure. Crude 30-day mortality after heart failure was slightly lower at the municipal hospitals than in the other types of hospitals. Patients at the regional hospitals also appear to be slightly younger than patients at other hospitals. Municipal hospitals had the highest percentage of Swedish patients, and regional hospitals had the highest percentage of non-Swedish patients. On average, the RS was similar in the three hospital types even if the percentage of patients in the higher RS group was slightly higher in the regional hospitals.

Table 2 shows principle causes of death for all patients with heart failure who died within 30 days of hospital discharge. Diseases of the circulatory system were the most common cause of death (51.6%), and within this category ischaemic heart disease (59.3%).

**Table 2:** Principal cause of death among the 3312 patients dying within 30 days after discharge from a Swedish hospital with a first ever diagnosis of heart failure during the period 2007–2009.

Principal cause of death	N	% of all deaths
Diseases of the circulatory system*	1709	51.6
- Ischaemic heart diseases	1014	59.3
- Other forms of heart disease	364	21.3
- Cerebrovascular diseases	134	7.8
- Hypertensive diseases	71	4.2
- Diseases of arteries, arterioles and capillaries	68	4.0
- Pulmonary heart disease and diseases of pulmonary circulation	34	2.0
- Chronic rheumatic heart diseases	18	1.1
- Diseases of veins, lymphatic vessels and lymph nodes, not elsewhere classified	4	0.2
- Other and unspecified disorders of the circulatory system	2	0.1
Neoplasms	552	16.7
Diseases of the respiratory system	329	9.9
Diseases of the digestive system	173	5.2
Endocrine, nutritional and metabolic diseases	122	3.7
External causes of morbidity and mortality	105	3.2
Certain infectious and parasitic diseases	93	2.8
Diseases of the nervous system, mental and behavioral disorders	85	2.6
Other	144	4.4

*The percentages for the sub-categories under diseases of the circulatory system are calculated considering as denominator the 1709 patients who have circulatory diseases as the principle cause of death.

### Measures of association and specific ward and hospital effects

Table 3 shows that, as expected, the RS was strongly associated with 30-day mortality (We show only the 1^st^, 4^th^, 7^th^ and 10^th^ decile groups, as it is sufficient to illustrate the association). Independently of the RS we additionally observed that non-Swedish females have a lower mortality risk than the Swedish females. The last model 4 even shows that, in the adjusted analyses, compared with the wards with the lowest volume of patients, patients treated at wards with the highest volume of patients have the lowest mortality risk, yet the POOR was rather high for the 3^rd^ category (POOR = 15%), and especially higher in the 2^nd^ category (POOR = 33%), which indicates the existence of considerable ward heterogeneity for this association.

Patients treated at municipal hospitals presented a somewhat lower mortality risk than those treated at the regional hospitals. However, the 95% CI included one and the POOR was 16%. In addition, on average, patients treated at the provincial hospitals had a slightly higher mortality and the POOR was only 3%.

**Table 3:** Measures of association (fixed effects) obtained by single (Models 1 and 2) and by three level (patients, wards and hospitals) multilevel logistic regression analysis (Models 3 and 4) modeling 30-day mortality after hospitalization from heart failure in patients who were admitted to Swedish hospitals (2007–2009). Values are odds ratios (OR) and 95% confidence intervals (CI) as well as Percentage of Opposite Odds Ratio (POOR).

	Single level models		Multilevel models	
	Model—1	Model—2	Model—3	Model—4
Risk score* (decile groups)				
- 1st	Reference	Reference	Reference	Reference
- 4th	3.67 (2.57–4.85)	3.58 (2.63–4.70)	3.70 2.69–5.06)	3.59 2.77–5.06)
- 7th	7.37 (5.49–9.85)	7.29 (5.49–9.53)	7.17 5.46–9.44)	7.06 5.52–9.59)
- 10th	24.48 (17.87–31.56)	23.81 (17.53–30.81)	22.99 (17.55–30.88)	22.24 (17.57–29.96)
Gender by Ethnic-origin group				
- Swedish Females		Reference	Reference	Reference
- Swedish Males		1.11 (1.02–1.19)	1.12 (1.02–1.21)	1.13 1.04–1.22)
- Non-Swedish Males		1.01 (0.89–1.17)	1.03 (0.88–1.19)	1.03 0.89–1.20)
- Non-Swedish Females		0.78 (0.66–0.92)	0.80 (0.67–0.96)	0.81 (0.69–0.98)
Ward volume of patients (tertile groups)**				
- 1st				Reference
- 2nd				0.81 (0.69–0.94)
				POOR = 33%
- 3rd				0.60 (0.48–0.76)
				POOR = 15%
Hospitals Classification				
- Regional				Reference
- Municipal				0.89 (0.75–1.07)
				POOR = 16%
- Provincial				1.26 (1.05–1.48)
				POOR = 3%

*Risk Score (RS) obtained from a logistic regression including age previous diagnoses (ICD-10) of diseases of the cerebral arteries (I6), arrhythmia (I48-I49), hypertension (I10-I13, I15), ischemic coronary artery disease (I20-I25), varicose (I83), peripheral vascular disease (I74, I80), acute myocardial infarction (I21), other types of heart disease (I3-I5), respiratory diseases (J0-J9), digestive diseases (K0-K9), diabetes (E10-E14), infectious diseases (A0-A9), cancer (C1-D4), lung cancer (C34), chronic diseases of the lower respiratory tract (J4), immunity disorder (D50-D89), mental diseases (F0-F9), and injury (S00-T14).

**Volume of patients with a diagnosis of heart failure (I50) treated at the ward.

### Measures of variance and general wards and hospital effects

Models 3 and 4 in Table 4 present the results of the variance components multilevel analysis. Both the ward and hospital components of variance were small. However, the ICC for the ward level was much higher (i.e. 5.3%) than the ICC of the hospital which was very close to zero (i.e., 0.4%). Also the MOR_W_ from models 3 and 4 were higher than the MOR_H_.

**Table 4:** Measures of area under the receiver operating characteristics curve (AUC) and measures of variance (random effects) obtained by Single Level Logistic Regression (Models 1 and 2) and Three Level (patients, wards and hospitals) Multilevel Logistic Regression (Models 3 and 4) modeling 30-day mortality after hospitalization from heart failure in patients who were admitted to Swedish hospitals (2007–2009). Values are median and 95% credible/confidence intervals (CI).

	Single level Models		Multilevel Models	
	Model 1	Model 2	Model 3	Model 4
AUC	0.727 (0.719–0.736)	0.729 (0.721–0.738)	0.753 (0.745–0.762)	0.752 (0.743–0.760)
Difference in AUC value	-0.002	Reference	0.024	0.023
Measures of Variance				
- Between hospitals			0.013 (0.001–0.049)	0.007 (0.001–0.052)
- Between wards			0.170 (0.117–0.241)	0.117 (0.066–0.174)
- PCV_H_			Reference	-46.15%
- PCV_w_			Reference	-31.18%
- ICC_H_			0.004 (0.000–0.014)	0.002 (0.000–0.015)
- ICC_w_			0.053 (0.035–0.081)	0.036 (0.020–0.064)
- MOR_H_			1.11 (1.03–1.23)	1.08 (1.03–1.24)
- MOR_w_			1.50 (1.39–1.67)	1.40 (1.28–1.57)
BDIC	20259.304	20211.665	19956.656	19960.730
Difference in BDIC	Reference	-47.639	-302.648	-298.574

Model 1: Single model with the individual Risk Score (RS). Model 2: model 1 plus gender by ethnic-origin group. Model 3: model 2 plus hospital and wards random effects. Model 4: model 3 plus ward volume of patients with heart failure, and hospital classification. PCV: Proportional Change in Variance. ICC: Intra-Class Correlation. MOR: Median Odds Ratio. BDIC: Bayesian Deviance Information Criterion.

**Fig 2:**
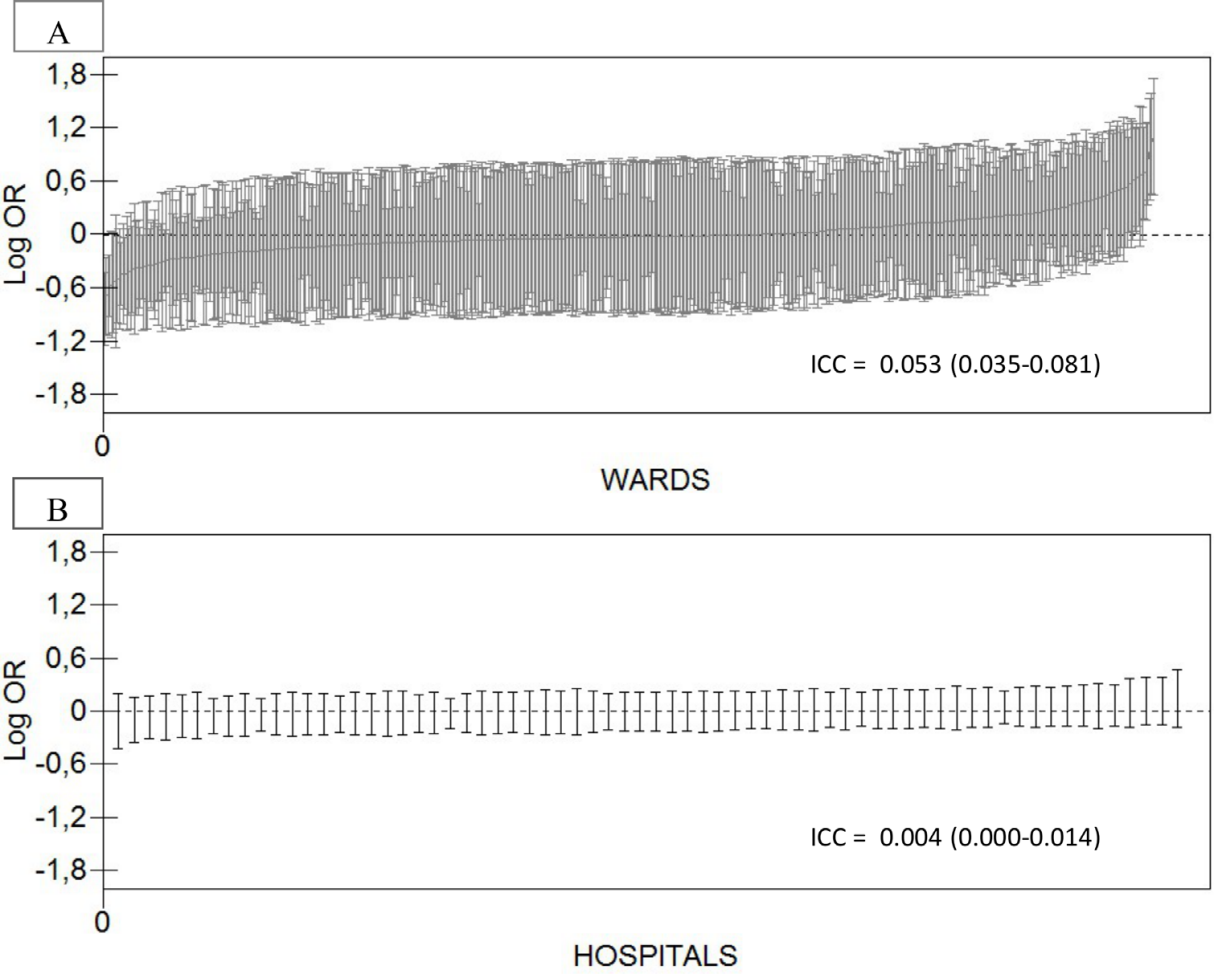
Ranking of the 71 hospitals (A) and 565 wards (B) according to their 30-day mortality after hospitalization for heart failure (2007–2009). Values are logarithm odds ratios (i.e., shrunken residuals) with 95% confidence intervals (vertical lines) adjusted for mortality risk score, sex and ethnic origin (see model 3 in Tables 3 and 4). The figure also indicates the values of the hospital and wards intra-class correlation coefficients (ICC) for 30-day mortality.

These findings need to be considered when interpreting Fig 2 (based on model 3 in Tables 3 and 4) which shows the ranking of the wards and hospitals according to their average short term mortality using the overall mortality risk as a reference. Besides the initial low general ward and hospital effects, there is a considerable uncertainty of the estimated averages, which expresses itself as a considerable overlapping of the 95% CIs.

The inclusion of the hospital and ward characteristics in model 4 of Table 4 explained about 46% of the minor hospital variance and about a third of the small ward variance.

### Measures of discriminatory accuracy (AUC)

The very inclusion of the RS in model 1 (Table 4) produced an AUC of 0.727 that did not increase substantially when including information on gender and ethnicity in model 2. The same was true when including the random intercepts for the ward and hospital levels.

**Fig 3:**
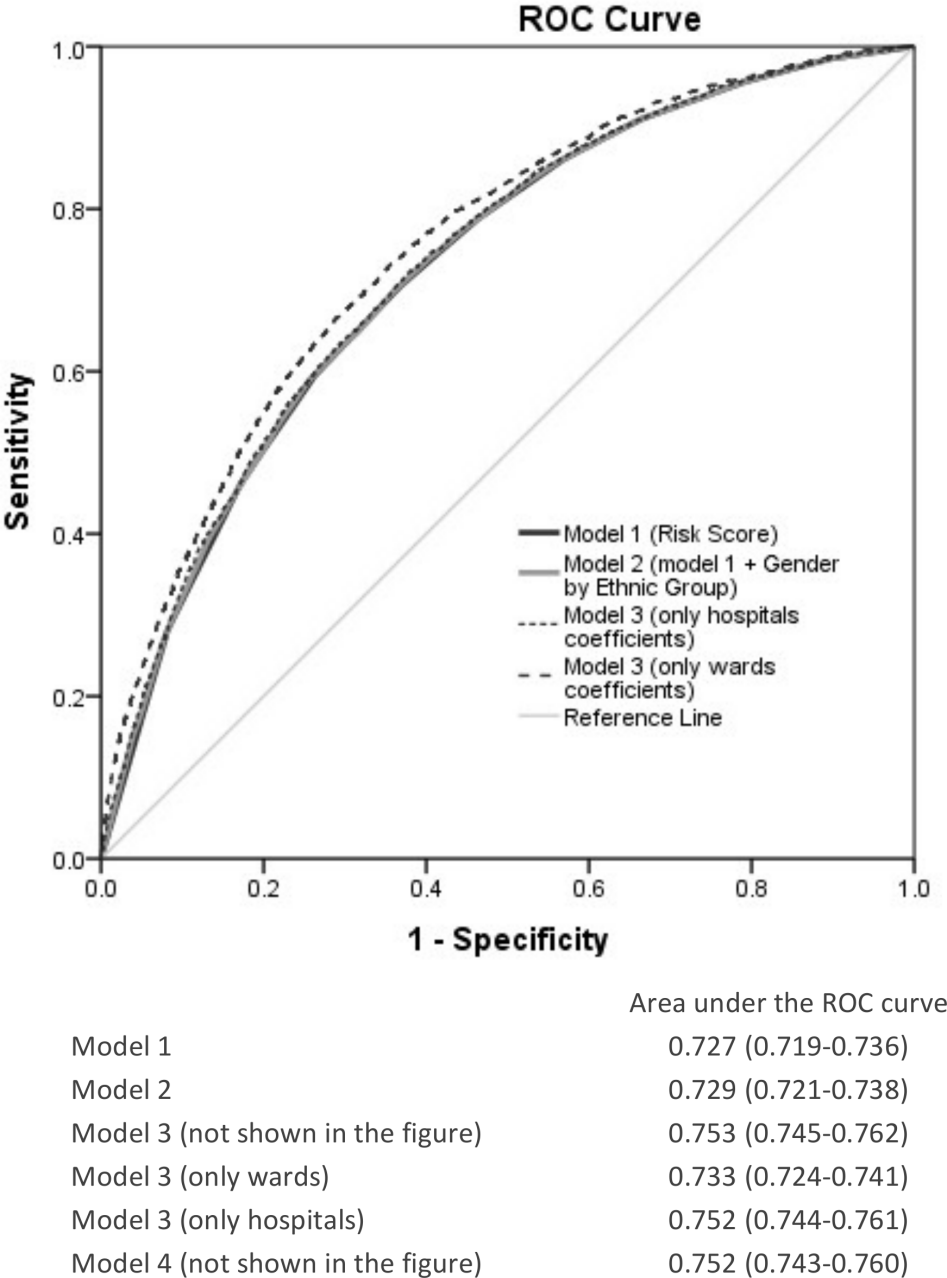
Receiver operating characteristics (ROC) curves and areas under the ROC curves (AUC) for the different models analyzed in the study. Model 1 (black line) is a simple logistic regression model including the individual risk score. Model 2 (grey line) is as model 1 but adding sex and ethnicity in categories. Model 3 is as model 2 but adding information on hospitals and wards in a multilevel logistic regression analysis. The ROC curve for model 3 is split showing the contribution of the ward level (thick dotted line) and of the hospital level (thin dotted line).

Fig 3 shows the ROC curves for the models distinguishing the separate contribution of the ward and hospital levels of model 3. It is clear that the (small) increase of the AUC for model 3 as compared with model 1 is due to the ward component. All the AUC values along with their 95% CIs are presented as part of Fig 3.

### Bayesian Deviance Information Criterion (BDIC)

The BDIC value for the first reference model considerably decreased in model 2 that included the sex and ethnicity of the patients. However, we observed the highest reduction in models 3 and 4, which incorporated the random intercepts for the ward and hospital levels (see Table 4).

## Discussion

### General study findings

We evaluated hospital performance in Sweden using an outcome indicator, 30-day mortality after heart failure. Beyond evaluating the hospital performance, our aim was to illustrate an innovative, stepwise-multilevel, logistic regression analysis of discriminatory accuracy recently developed by this team [31]. This methodological approach combines single and multilevel regression models in order to evaluate the possible existence of general, unspecified institutional (i.e., wards and hospitals) effects which could condition individual prognosis over and above individual characteristics. Such observational effects are quantified by measures of clustering like the ICC [2, 10, 32], heterogeneity measure MOR [41], as well as by measures of discriminatory accuracy like the AUC [29–31].

We found that, overall in Sweden, 30-day mortality after heart failure was around 9%. This percentage appears rather low as comparing with other countries such as the United States with national values around 11–12% (2008–2012) including USA Medicaid hospitals with values between 10.8–11.29% (2008–2011) [45], but still higher than the 6.6–7.9% (2005–2008) [5] and 7.5–8.2% (2008–2009) [46] benchmarking values reported by some studies and hospitals in the United States. However, those figures are not fully comparable since there may be population differences in age structure, co-morbidities as well as non-standardized diagnostic criteria needing special consideration when comparing different health care settings.

We also observed differences between hospitals and, especially, between wards in average 30-day mortality around the national average of 30-day mortality. However, those differences between averages provide insufficient information that cannot be used for discriminating patients who will survive or not. Rather than differences between averages, what matters is to know the share of the individual differences in the propensity of dying that are at the hospital level. From this perspective, the almost non-existent hospital ICC (i.e., 0.04%) suggests that all hospitals in Sweden had a similar performance.

It is obvious that each individual hospital may have its own context that embraces factors like internal organization, treatment guidelines and harmonized clinical practices that could condition patient survival over and above patient characteristics. That is, the same patient would have a different prognosis if she/he was treated at a specific hospital rather than another. If this is true, we should expect a large general hospital effect that expresses itself as a high ICC. Also, the AUC would be expected to increase considerably after adding the hospital level to the predictive equation. Contrary to this, our results indicate that the Swedish hospitals were like random samples from the whole population of patients with heart failure. The ranking of hospitals by a so-called *league table* (Fig 2B) shows that no one of the hospitals could be distinguished with certainty from the overall average mortality. In addition, the hospital ICC was close to null and the AUC analysis confirmed the interpretation of the ICC values. This demonstrates that knowledge of patients age and previous hospitalizations was enough to obtain a relatively high discriminatory accuracy (AUC = 0.727). Further information on a patient sex or ethnicity only resulted in a minor contribution and the same was true when adding information on the hospital or ward where the patient was treated. However, the hospital level hides some ward heterogeneity. In fact, despite that the ward ICC was small (i.e., 5.3%), it was much higher than the hospital ICC.

In practice, our results suggest that any intervention directed at decreasing 30-day mortality in patients with heart failure should be focused on all Swedish hospitals and not only on those with an average mortality higher than the national average. We could consider launching an intervention in specific wards (i.e., those with the highest mortality in Fig 2A) but again, neither the ward ICC nor the AUC analysis gives strong support for this initiative. Therefore, despite findings from traditional measures of association (OR and 95% CI) indicating that wards with higher patient volumes, and municipal hospitals shown lower average mortality, we cannot qualify these wards and hospitals as better performers since the discriminatory accuracy of the ward and, more so, the hospital level was very low. Furthermore, the lower mortality risk found in municipal hospitals compared with regional hospitals was not conclusive and the POOR was 16% suggesting the existence of a weak and heterogeneous pattern of association. Even if patients treated at the provincial hospitals had a slightly higher mortality and the POOR was only 3%, the initial general hospital effect was very low, which makes this finding rather irrelevant.

In summary, our study shows that we cannot count on traditional measures based on differences between averages to evaluate hospital performance. The multilevel methodological approach we are promoting [31] needs a joint analysis including patient variables, hospital/ward units, and hospital/ward specific characteristics. The traditional use of league tables, funnel plots or reporting “significant” hospital variance alone should be reconsidered. Rather, a proper evaluation of hospital performance needs to include measures of association, variance and discriminatory accuracy.

The study includes three analytical steps. The first step (models 1 and 2) analyzes patient-level covariates in standard (i.e., single-level) logistic regressions. The selection of these individual variables is based on the assumption that they are confounders (i.e., patient mix).The second step (model 3) quantifies general hospital effects by measuring the ICC, the MOR and the increment in the AUC obtained by adding hospital/ward level information. We only include the hospital/ward codes without specifying any hospital/ward characteristics. The final step (Model 4) includes specific hospital/ward information (ward volume and type of hospital). In this model, the interpretation of the OR, and the POOR must always be done in relation to the hospital/ward variance ($\sigma_{h}^{2}\;and\;\sigma_{w}^{2}$.) obtained in step 2 (Model 3) and the PCV associated with moving from model 2 to model 3. For instance, suppose Model 3 estimated a high value for $\sigma_{h}^{2}$ and therefore a high ICC_h_ for the binary outcome “30-day mortality”. Thereafter, in Model 4, we include contextual variables (e.g., type of hospital). If, for instance, the type of hospital is associated with the outcome (a high OR) and it explains a large share of $\sigma_{h}^{2}$ (PCV is high) the POOR would be low. This case illustrates a situation where the hospital context conditions the mortality (i.e., high $\sigma_{h}^{2}\;\sigma_{u}^{2}$ and ICC_h_). It also demonstrates that this influence appears mediated by the contextual variable (type of hospital) so the hospital variable is not only strongly associated with the outcome, but it also explains the hospital variance and thereby shows a low POOR. In other words, the conclusion would be that the hospital context influences the individual mortality and that this influence has to do with the type of hospital. However, there are other possible situations. For instance, $\sigma_{h}^{2}$ could be very low from the beginning (Model 3) and the hospital variable could be significantly associated to the outcome but still does not explain much of the $\sigma_{h}^{2}$ (i.e., low PCV) in Model 4. Nevertheless, since $\sigma_{h}^{2}$ was low from the beginning, the POOR would be low. In this case, the hospital context would have a small influence on patient mortality even if the variable hospital type is, on average, associated with mortality and the POOR is low. Our results are included in this last scenario.

### Strengths and weaknesses

This study has a number of strengths. The Swedish registers have a considerable quality and they are based on standardized procedures for data collection and storage [47]. As the follow up only concerned the first 30 days after discharge from the hospital, the follow up data for short term mortality is, in principle, complete. Initially, we used data on all patients who met the inclusion criteria and were admitted to Swedish hospitals between 2007 and 2009. The registers cover the entire country of Sweden and information on mortality is recorded even when a Swedish resident dies outside the country.

We did, however, include Swedish hospitals that admitted at least 50 patients, which may have introduced some selection bias. This reduced extrapolation on the hospital specific associations obtained in MLRA. However, variance is not misestimated by this procedure since in MLRA the variance concerns the shrunken residuals and small size hospitals are shrunken towards the mean to avoid statistical noise.

To adjust the difference in patient-mix we covered a wide range of co-morbidities which are known in literature to be related to mortality after HF[48, 49].Additionally, we used a RS [50] [51] which reduced the number of predictors, and the number of outcome events per variable was large enough [52].

On the other hand, this study has specific limitations. The models employed lack information on previous and current medications, as well as life style indicators on obesity, smoking and alcohol consumption [48, 49]. Additionally, no model validation was performed. Yet, if the models were over fitted, our prediction would be an overestimation so the small contribution of the hospital and ward components would even be lower. Second, the purpose of our study was not primarily to create a new risk score equation but rather to adjust for patient mix. Although, the analytical methodology we propose can be implemented in many diverse contexts, yet the results of our study concern only Swedish hospitals and wards and the RS equation we obtained cannot be used for prediction outside Sweden [51].

### Other studies

The use of ROC plots and the calculation of AUC are common practices for the evaluation of model fitting in health care epidemiology, and this approach has been criticized in a previous study by Katzan et al. 2014 [53] p. 922. These authors concluded that "An important limitation of the C statistic is that it only measures the predictive power of a model at the patient level and does not directly express the ability of the model to accurately profile hospitals with respect to the hospital specific risk-standardized ratios". However, our approach actually gives a solution to those critics as the quantification of hospital general effects allows to accurately profile hospitals. For this purpose, we can calculate the hospital ICC adjusted for patient variables (i.e., Model 3 in our study) or perform a two-step analysis using the AUC. That is, first we fit a customary single level logistic regression model only including individual characteristics, thereafter, we use a multilevel regression model that includes a random effect for the hospital (and wards) level and, finally, we calculate the improvement in the AUC that is due to the general hospital effect. For more information on the link between AUC and ICC and particularities of each measure, please see S1 Appendix.

As far as we known, vast majority of previous studies have primarily used the AUC or C-statistic solely to statistically check the model performance using fixed effect analyses ignoring the multilevel nature of the variance.[54–57]. On some occasions, researchers have applied mixed effect models considering the multilevel structure of the data [58, 59], but they have not used the AUC for evaluating hospital general effects as this study does. Those multilevel studies did not explicitly consider the AUC to quantify if the hospital/ward level information could discriminate between patients who will and who will not suffer the outcome as is done in the current and in previous studies [29, 30, 60]. As far we know, the AUC approach for evaluating hospital performance was introduced by this team [29, 30] and was followed by a French publication.[61]

In essence, the idea of using measures of variance like the ICC [2, 10] for the evaluation of institutional performance is not new and this team already applied it to evaluate survival after initial hospitalization for heart failure [2] and myocardial infarction [62] in Sweden in 2001. Using this methodology we also audited neonatal mortality in the Swedish acute care hospitals [9] and evaluated different quality indicators for medication use in the country [10]. We have applied a similar approach when it comes to investigate geographical differences in health outcomes [63] [22] [64] [65]. The methodology we use has also been recommended in a white paper published in 2011[26] by the committee assigned to set statistical guidelines for assessing Centers for Medicare and Medicaid Services (CMS) hospital performance in USA. S1 Appendix incorporates more details on estimating ICC, a conceptual link between ICC and AUC, as well as alternative measures of clustering.

We agree with Krumholz *et al*, *2013* [66] in that *for evaluating hospital quality*, *we are seeking to measure a latent variable of quality*. This idea approximates the concept of general (or “latent”) hospital effect in our model 3 and the use of the ICC we have promoted in this and previous studies of ours [25, 67]. However, the innovativeness of our study is that by including the hospital residuals from the MLRA in the prediction and calculating the AUC we are able to quantify general (i.e., “latent”) hospital effects in the same way we evaluate the predictive ability of patient level variables [29, 30].

Observe that the hospital variance can be very small but still “statistically significant” if the sample is large. What matters most is not statistical significance but the size of the ICC. This idea can also be expressed by the AUC approach discussed in this study.

### A simple way of evaluating performance using multilevel analysis of discriminatory accuracy

Two sources of information are needed to evaluate hospital and ward performance: 1) the overall average of short term patient mortality in Sweden, and 2) the variation around this average. The multilevel analysis decomposed this variance at the hospital, ward and patient level, which allows us to identify which of those levels is actually relevant for the outcome. With regard to this study, it is important to stress that the low ICC/AUROC by itself does not mean that the hospital level has no influence on patient’s survival. It is obvious that the care and treatment of heart failure patients at the hospital conditions their prognosis. A low ICC/AUC means there are no remarkable hospital differences. In other words, information at hospital and ward levels does not contribute to understanding individual differences in short term mortality after admission with heart failure. For instance, an explanation could be that all hospitals are performing homogeneously in Sweden.

In practice, we could imagine four extreme situations combining overall prevalence (good vs. bad) and measures of ICC/AUROC (low vs. high) with gliding situations between them. We formulate these scenarios for the hospital level. If the overall survival is good (i.e., high) and the ICC low, all hospitals were doing homogeneously well. However, if the overall survival is low and the ICC low, all hospitals were doing homogeneously bad. In practice, a low ICC suggests that we do not need to point out specific hospitals to improve patient survival. Rather, the focus on intervention should be targeting all hospitals in the country. However, the higher the ICC the more appropriate to act on the hospitals with the worst survival. A similar reasoning can be applied when using the AUC in combination with the overall average 30-day mortality. If the hospitals and wards do not add discriminatory accuracy, it would not be effective to focus on certain hospitals. If we aim to decrease mortality, we should focus on improving care in the patients with higher mortality while it does not matter which is the hospital of treatment. In our study, knowing the patient age and previous diseases (i.e., the RS) was enough to achieve an AUC = 0.727 which was only slightly better when knowing the sex and ethnic status of the patient (+0.002 units). However, this patient level information was only marginally improved by knowing the hospital or the ward where the patient was treated.

## Conclusions

In summary, we may find a good or a bad overall average for the quality indicator but before planning an intervention at the actual institutional level (e.g., hospitals, wards) we need to consider other complementary sources of information such as the size of the ICC or AUC or both depending on the purpose of evaluation. As it concerns 30-day survival after HF in Sweden, we observed that the Swedish hospitals were performing homogeneously well. However, to further increase survival after HF, it is necessary to improve treatment in high risk patients regardless of which hospital they are treated in.

## Supporting Information

S1 Appendix Supplementary Methodological Section. (DOCX)
