# Supplementary material for: Short Term Survival after Admission for Heart Failure in Sweden: Applying Multilevel Analyses of Discriminatory Accuracy to Evaluate Institutional Performance
Source: PLoS One. 2016 Feb 3;11(2):e0148187. doi: 10.1371/journal.pone.0148187 (PMC4739586; doi:10.1371/journal.pone.0148187)
Supplement: S1 Appendix — (DOCX) [file pone.0148187.s001.docx]

**S1 Appendix. Supplementary Methodological Section**

**Mathematical Equations for the Fitted Models**

The first model (*model 1*) is a single level logistic regression model including only the risk score for mortality (RS).

$y_{i} \sim Binomial \left( \pi_{i} \right)$

$logit \left( \pi_{i} \right)= \beta_{0i}+\beta_{1i}RS$  *Formula 1*

Where $\beta_{0i}$ is the intercept and $\beta_{1i}$ is a set of coefficients for the RS

In the second model (*model 2*), we added the combined explanatory variable of gender by migration/ethnic status.

$y_{i} \sim Binomial \left( \pi_{i} \right)$

$logit \left( \pi_{i} \right)= \beta_{0i}+\beta_{1i}RS$ $+\beta_{2i}gm$  *Formula 2*

Where $\beta_{2i}gm$ is a set of regression-coefficients for the dummy variables of combined gender and ethnic status.

Then, in the next model (*model 3*), we expanded model 2 by including two random intercepts: one for the wards inside hospitals and another for the hospitals level in a three level multilevel regression model.

$y_{i} \sim Binomial \left( \pi_{i} \right)$

$logit \left( \pi_{i} \right)= \beta_{0h}+\beta_{0w}+\beta_{1i}RS$ $+\beta_{2i}gm+u_{0h}$ *+*$u_{0w}$ *Formula 3*

Where$\beta_{0h}$ and $\beta_{0w}$ are the hospital (h) and ward (w) intercepts and $u_{0h}$ and $u_{0w}$ are their respective residuals that are normally distributed with mean 0 and variances$\sigma_{h}^{2} \mathrm{and}\sigma_{w}^{2}$.

From this model *(Model 3),* we calculated the intra-class correlation coefficient for the hospital level (ICCh) as:

ICC_h_$=\frac{\sigma_{h}^{2}}{\left( \sigma_{h}^{2}+\sigma_{w}^{2}+\frac{\pi^{2}}{3} \right)}$ *Formula 4*

Where $\sigma_{h}^{2}$is the hospital level variance, $\sigma_{w}^{2}$ is the ward variance and $\frac{\pi^{2}}{3}$ denotes the variance of a standard logistic distribution. (Note that here $\pi$ denotes the mathematical constant 3.1416…, not the probability.).[1].

And similarly we computed the ICCw for the ward as

ICC_w_$=\frac{\sigma_{h}^{2}+\sigma_{w}^{2}}{\left( \sigma_{h}^{2}+\sigma_{w}^{2}+\frac{\pi^{2}}{3} \right)}$ *Formula 5*

The ICC_h_ expresses the correlation in the propensity of dying between two patients taken randomly from the same hospital while the ICC_w_ indicates the correlation in the propensity of dying between two patients taken randomly from the same ward which obviously are from the same hospital.

Further, we calculated the Median Odds Ratio for hospital level as:

MOR_h_ ≈ $\exp\left( 0.95\times\sqrt{\sigma_{h}^{2}} \right)$ *Formula 6*

Analogously the MOR_w_ for the ward was:

MOR_w_ ≈ $\exp\left( 0.95\times\sqrt{\sigma_{h}^{2}+\sigma_{w}^{2}} \right)$ *Formula 7*

Even if both the ICC and the MOR are based on the same hospital/ward variance, $\sigma_{u}^{2}$ , the MOR is conceptually a measure of heterogeneity, rather than of clustering as the ICC defined earlier in the paper. The MOR is an alternative way of expressing hospital/ward variance from a probabilistic perspective. The MOR translates the hospital/ward variance to the widely used OR scale, which makes the MOR comparable with the OR of individual or hospital/ward variables. The MOR is defined as the median value of the distribution of ORs obtained when randomly picking two individuals from different hospitals/wards, and comparing the one from the highest risk hospital/ward to the one from the lowest risk hospital/ward. In simple terms, the MOR could be interpreted as the increased (median) odds of dying if an individual moves to another hospital/ward with a higher risk.

In the next model 4 we added the ward and the hospital level specific variables indicating the volume of patients with heart failure (in tertile groups) as well as the hospital classification.

$y_{i} \sim Binomial \left( \pi_{i} \right)$

$logit \left( \pi_{i} \right)= \beta_{0h}+\beta_{0w}+\beta_{1i}RS$ $+\beta_{2i}gm{+\beta}_{3j}{np}_{ward}{+\beta}_{4j}HC+ u_{0h}+u_{0w}$ *Formula 8*

Where $\beta_{3j}{np}_{ward}$ and $\beta_{4j}HC$ are sets of regression-coefficients for the dummy variables of tertiles of number of patients at the ward (np_ward_) and hospital classification (HC).

**The Proportion of Opposed Odds Ratios (POOR)**

As in the case of individual-level observational effects like the association between RS and mortality, specific contextual effects of the hospitals and wards are estimated using measures of average effect such as ORs and 95% confidence intervals, although we aimed to avoid a common misconception associated with carrying multilevel regression analyses which gives a “population average” interpretation to the OR of contextual variables [2-4]. We need to consider that multilevel regression provides regression coefficients for individual patient variables that are adjusted for the hospitals and wards level random effects. That is, they reflect the association between patient level variables and the 30-day mortality within a specific cluster (i.e., hospital and ward). They are, therefore, termed ‘‘cluster specific” ORs. However, in multilevel logistic regression, a contextual OR can hardly be interpreted in this way since a hospital or ward variable is constant for all individuals in the cluster. To solve this situation, Larsen *et al* [2, 3] suggested including the cluster variance in the quantification of a contextual OR. One method of doing this is to calculate the Proportion of Opposed Odds Ratios (POOR) i.e., the proportion of ORs with the opposite sign to the overall OR [4]. For our binary measure of 30-day mortality, the POOR for the hospital variable is calculated as:

$POOR=\Phi\left( -\frac{\beta_{4j}}{\sqrt{2\sigma_{h}^{2}}} \right)$ *Formula 9*

And for the POOR the ward variable is calculate as

$POOR=\Phi\left( -\frac{\beta_{3j}}{\sqrt{2(\sigma_{h}^{2}+\sigma_{w}^{2}}} \right)$. *Formula 10*

**Discussion of the Methods**

It is worth noting that we did not perform internal validation of the models as this practice is not recommended for prediction modeling studies by the CHARMS Guidelines [6]. For instance, randomly splitting the dataset into two datasets and using one for developing the models and the other one for validating the models is not as useful as it tends to produce very similar results while it reduces the volume of the used dataset. Alternatively, other techniques such as  [random sampling with replacement](http://en.wikipedia.org/wiki/Random_sampling_with_replacement) (i.e., bootstrapping) or using a non-random split of the dataset by region, hospitals or time [6] could have been applied but, as mentioned earlier in the paper, the aim of our study was not to create a new equation for prediction but, rather, to quantify the general hospital general effects in the temporal-spatial context of our study.

A classical question when investigating differences between contexts like hospitals or small geographical areas is “What is too much variation?” [23] . The multilevel, analytical approach gives a clear answer [14] since it partitions the *total* individual variance into different levels (i.e., individual variance within hospitals and individual variance between hospitals) so a large hospital variance means that the hospital level accounts for a large share of the total individual variance.

In essence, the concept of ICC is easy to understand when analyzing continuous, normally distributed variables. However, for binary variables in generalized, linear multilevel models, the individual and the contextual level variances are at different scales which cause technical difficulties for the calculation and interpretation of the ICC [1]. This problem is well known in the multilevel analysis literature and there are today valid procedures for estimating the ICC for binary outcomes, such as: the normal approximation, the simulation method, Taylor series linearization, and the latent variable threshold approach [1, 4, 17, 18]. In addition to these, there are alternative measures of clustering like the pairwise odds ratio (PWOR) [19] and measures of heterogeneity like the median odds ratio (MOR) [2, 3] which also quantify general contextual effects. Interestingly, discriminatory accuracy can be quantified using the *tetrachoric correlation* [20, 21] which conceptually links the ICC and the AU-ROC as measures of general hospital effects. A low ICC for binary outcome can be interpreted as in the continuous case. However, when the prevalence of the outcome is extreme (i.e., very low or very high), the hospital variance, and thereby the ICC, needs be computed using a probit rather than a logit link. In the probit link the variance of the latent individual variable is normally distributed and equal to 1.[22]

There is a strong similarity between the ICC and the AU-ROC, so when the ICC is high the AU-ROC is also high. However, the ICC is not influenced by the number of patients at the hospital as its calculation is based on the hospital variance which, in turn, is based on differences between hospital averages and it is, consequently, standardized for hospital size (i.e., the number of patients in the hospital). Alternatively, the AU-ROC is based on the calculation of the TPF and FPF for different thresholds of the predicted probability. Since this predicted probability is an individual level variable, large hospitals contribute with more individuals (patients). Because of this difference, it would be possible to find a high ICC but a low AU-ROC (which was not the case in our study) if the number of individuals is relatively much larger in some hospitals than in others. This situation does not mean that the AU-ROC is a biased measure but, rather, it provides different and useful information. For instance, some large hospitals could have a *high* proportion of patient death within 30 days and some small hospitals could have a *low* proportion. The ICC would be high indicating that hospitals condition individual mortality. However, the AU-ROC would be low expressing that most patients have the same predicted risk, irrespective of whether they die or not, and subsequently, that information on hospitals do not discriminate with accuracy the patients who die from those who survive. Otherwise, when the sizes of the hospitals are similar there is a clear correspondence between the ICC and the AU-ROC values [7, 8].

We used hospitals residuals from models 3 and 4 for the calculation of the corresponding AU-ROC, however − to some extent − an alternative to this approach would be to include the hospitals as dummy variables in a single level logistic regression (i.e., “fixed effects approach”) and calculate the increment in the AU-ROC compared to a model that only includes patient characteristics (i.e., patient-mix). This represents a convenient tool for a quick estimation of general contextual effects (i.e., hospital and ward effects). Yet, this approach has other limitations [24]. The fixed effects (i.e., with dummy variables for hospitals) approach hinders our ability to study specific contextual effects (i.e., ward volume of patients, and hospital classification). Furthermore, inclusion of the wards/hospitals as dummy variables explains all between ward/hospital variance, while the multilevel approach allows investigating which characteristics of the ward/hospital can explain the ward/hospital heterogeneity. Besides, a fixed effects model is not parsimonious, as we would need 70 dummy variables for the 71 hospitals and another 564 variables for the 565 wards to be included in the single level regression model instead of only two random intercepts for wards/hospitals in the multilevel models. The fixed effects approach is also susceptible to biased estimation of hospital averages by random noise if the number of patients in some wards/hospitals is small, in contrast to the multilevel regression as the outcome prediction is based on shrunken residual [25] that takes into account the information available at each ward/hospital.

**References**

1. Goldstein, H., W. Browne, and J. Rasbash, *Partitioning variation in multilevel models.* Understanding Statistics, 2002. **1**: p. 223-32.

2. Larsen, K., et al., *Interpreting parameters in the logistic regression model with random effects.* Biometrics, 2000. **56**(3): p. 909-914.

3. Larsen, K. and J. Merlo, *Appropriate assessment of neighborhood effects on individual health: integrating random and fixed effects in multilevel logistic regression.* Am J Epidemiol, 2005. **161**(1): p. 81-8.

4. Merlo, J., et al., *A brief conceptual tutorial of multilevel analysis in social epidemiology: using measures of clustering in multilevel logistic regression to investigate contextual phenomena.* J Epidemiol Community Health, 2006. **60**(4): p. 290-7.

5. Ludvigsson, J.F., et al., *External review and validation of the Swedish national inpatient register.* BMC Public Health, 2011. **11**: p. 450.

6. Moons, K.G.M., et al., *Critical Appraisal and Data Extraction for Systematic Reviews of Prediction Modelling Studies: The CHARMS Checklist.* PLoS Med, 2014. **11**(10): p. e1001744.

7. Wagner P and Merlo J, *Measures of discriminatory accuracy in multilevel analysis.* European Journal of Epidemiology, 2013. **28**(1, Supplement): p. 135.

8. Wagner P and Merlo J, *Discriminatory accuracy of a random effect in multilevel logistic regression.* 20th IEA World Congress of Epidemiology (WCE2014). 2014.

9. Merlo, J., et al., *Survival after initial hospitalisation for heart failure: a multilevel analysis of patients in Swedish acute care hospitals.* J Epidemiol Community Health, 2001. **55**(5): p. 323-9.

10. Ohlsson, H., et al., *Performance evaluations and league tables: do they capture variation between organizational units? An analysis of 5 Swedish pharmacological performance indicators.* Med Care, 2011. **49**(3): p. 327-31.

11. Merlo, J., et al., *[Multilevel analysis of regional disparities in survival after heart failure: differences between county health services affect little patients' prognosis].* Lakartidningen, 2001. **98**(44): p. 4838-44.

12. Merlo, J., et al., *Hospital level of care and neonatal mortality in low- and high-risk deliveries: reassessing the question in Sweden by multilevel analysis.* Med Care, 2005. **43**(11): p. 1092-100.

13. Lynch, K.F., et al., *Context and disease when disease risk is low: the case of type 1 diabetes in Sweden.* J Epidemiol Community Health, 2010. **64**(9): p. 789-95.

14. Merlo, J., et al., *Bringing the individual back to small-area variation studies: a multilevel analysis of all-cause mortality in Andalusia, Spain.* Soc Sci Med, 2012. **75**(8): p. 1477-87.

15. Merlo, J., *Multilevel analytical approaches in social epidemiology: measures of health variation compared with traditional measures of association.* J Epidemiol Community Health, 2003. **57**(8): p. 550-2.

16. Merlo, J., *Invited commentary: multilevel analysis of individual heterogeneity-a fundamental critique of the current probabilistic risk factor epidemiology.* American Journal of Epidemiology, 2014. **180**(2): p. 208-12.

17. Browne, W.J., et al., *Variance partitioning in multilevel logistic models that exhibit overdispersion.* Journal of the Royal Statistical Society: Series A (Statistics in Society), 2005. **168**(3): p. 599-613.

18. Li J, Gray BR, and Bates DM, *An Empirical Study of Statistical Properties of Variance Partition Coefficients for Multi-Level Logistic Regression Models.* Communications in Statistics - Simulation and Computation, 2008. **37**: p. 2010–2026.

19. Petronis, K.R. and J.C. Anthony, *A different kind of contextual effect: geographical clustering of cocaine incidence in the USA.* J Epidemiol Community Health, 2003. **57**(11): p. 893-900.

20. Bennett, B., *Use of the tetrachoric correlation model in assessing clinical tests.* Biometrical Journal, 1980. **22**(4): p. 335–338.

21. Lorenzo-Seva, U. and P.J. Ferrando, *TETRA-COM: a comprehensive SPSS program for estimating the tetrachoric correlation.* Behav Res Methods, 2012. **44**(4): p. 1191-6.

22. Hosmer Jr, D.W., S. Lemeshow, and R.X. Sturdivant, *Applied logistic regression*. 2013: John Wiley & Sons.

23. Diehr, P., et al., *What is too much variation? The null hypothesis in small-area analysis.* Health Serv Res, 1990. **24**(6): p. 741-71.

24. Merlo, J., *Changing analytical approaches in European epidemiology -- a short comment on a recent article.* Eur J Epidemiol, 2005. **20**(8): p. 737; author reply 738.

25. Merlo, J., et al., *A brief conceptual tutorial of multilevel analysis in social epidemiology: linking the statistical concept of clustering to the idea of contextual phenomenon.* J Epidemiol Community Health, 2005. **59**(6): p. 443-9.
